# Artificial Citrate Operon Confers Mineral Phosphate Solubilization Ability to Diverse Fluorescent Pseudomonads

**DOI:** 10.1371/journal.pone.0107554

**Published:** 2014-09-26

**Authors:** Hemanta Adhikary, Paulomi B. Sanghavi, Silviya R. Macwan, Gattupalli Archana, G. Naresh Kumar

**Affiliations:** 1 Department of Biochemistry, Faculty of Science, The Maharaja Sayajirao University of Baroda, Vadodara, Gujarat, India; 2 Department of Microbiology and Biotechnology Centre, Faculty of Science, The Maharaja Sayajirao University of Baroda, Vadodara, Gujarat, India; Imperial College London, United Kingdom

## Abstract

Citric acid is a strong acid with good cation chelating ability and can be very efficient in solubilizing mineral phosphates. Only a few phosphate solubilizing bacteria and fungi are known to secrete citric acids. In this work, we incorporated artificial citrate operon containing NADH insensitive citrate synthase (*gltA1*) and citrate transporter (*citC*) genes into the genome of six-plant growth promoting *P. fluorescens* strains *viz*., PfO-1, Pf5, CHAO1, P109, ATCC13525 and Fp315 using MiniTn7 transposon gene delivery system. Comprehensive biochemical characterization of the genomic integrants and their comparison with plasmid transformants of the same operon in M9 minimal medium reveals the highest amount of ∼7.6±0.41 mM citric and 29.95±2.8 mM gluconic acid secretion along with ∼43.2±3.24 mM intracellular citrate without affecting the growth of these *P. fluorescens* strains. All genomic integrants showed enhanced citric and gluconic acid secretion on Tris-Cl rock phosphate (TRP) buffered medium, which was sufficient to release 200–1000 µM Pi in TRP medium. This study demonstrates that MPS ability could be achieved in natural fluorescent pseudomonads by incorporation of artificial citrate operon not only as plasmid but also by genomic integration.

## Introduction

Phosphorous (P) is one of the major nutrients limiting plant growth. Soluble P-ion concentration in most soils varies from 0.1 to 10 µM while P required for optimal growth ranges from 1 to 60 µM [1]. 70% of the phosphate in soils is present in complexes with cations, which is unavailable to plants. Rock phosphates are converted into soluble forms by using strong inorganic acids and provided for increased plant growth and crop yields. However, there is gradual and rapid depletion of global high-grade P resources, which could pose a major challenge for sustaining the agricultural productivity [2]–[4]. Increasing the efficiency of P application and use of low grade P reserves could delay the depletion of phosphate reserves. Phosphate solubilization using microorganisms has been proposed to be effective for this purpose. However, variable efficacies in field conditions have become a major hurdle in harvesting their potentials [5]. Soil properties, nature of rock phosphate, nature of the root exudates, carbon catabolite repression and presence of plasmid are known to decrease the effectiveness of phosphate solubilizing microorganisms (PSMs). However, secretion of strong organic acids by native rhizobacteria results in higher efficacy of PSMs in field conditions [6].

The secretion of gluconic acid by direct oxidation of glucose is the major mechanism of P-solubilization by most Gram-negative bacteria [7]. Gluconic acid biosynthesis is carried out by periplasmic glucose dehydrogenase (GDH) enzyme involving pyrroloquinoline quinone (PQQ) as the co-factor. Incorporation of genes involved in PQQ biosynthesis and transport in non-PSMs resulted in increased gluconic acid secretion and mineral phosphate solubilizing (MPS) ability [8], [9]. However, effectiveness of their MPS ability using gluconic acid is limited by the amount of aldose sugars naturally present in the root exudates. One potential solution for this problem is using stronger organic acids for P solubilization. For instance, in alkaline vertisol soils, P release is dependent on the nature and amount of organic acids in the order Acetic  =  Succinic  =  Lactic <<Gluconic <<Oxalic <Tartaric  =  Citric [10]. Thus, rhizobacteria secreting citric acid could be more effective in solubilizing P in alkaline vertisols. An ideal rhizobacteria that can be employed for this purpose is fluorescent pseudomonads which possess very good plant growth promotion abilities including biocontrol againstbacterial and fungal plant pathogens by secretion of a variety of compounds [11]. Hence, fluorescent pseudomonads producing citric acid could be very effective as P biofertilizers in alkaline soils.

Previously, our studies demonstrated accumulation of high levels of citric acid but low secretion upon heterologous overexpression of phosphoenolpyruvate carboxylase (*ppc*) and citrate synthase (*cs*) genes in *Pseudomonas fluorescens* ATCC13525 [12], [13]. Overexpression of these genes independently and together showed that CS activity chiefly regulates citric acid biosynthesis. CS enzymes in *E. coli* and *Pseudomonas* sp. are strongly inhibited by NADH [14]–[16]. Secretion of low citric acid, in spite of high intracellular citrate, suggested that native H^+^ dependent citrate transporter is not effective in citrate efflux, though it is very efficient in citrate uptake as reflected in terms of supporting the growth of the *P. fluorescens* ATCC 13525 on citrate as sole carbon source. Thus citric acid secretion could be dependent on the amount of intracellular citrate as well as the nature of citrate transporters. Citrate utilization in *Salmonella typhimurium* is mediated by Na^+^ dependent transporters (*citC*) belonging to hydroxy carboxylate transporter family [17]. Citrate in complex with Mg^2+^ is the major citrate-uptake system (CitM) in soil bacterium *Bacillus subtilis* during growth on citrate under aerobic conditions [18], [19]. The CitC and CitM are best characterized transporters used for citrate utilization. These transporter proteins are involved in pseudo-symmetrical transport, can alternate between atleast two preferred conformations [20], [21]. Functional characterization and structural determination of the Na^+^ dependent dicarboxylate transporter VcINDY from *Vibrio cholera* also revealed the possible reversibility [22]. Therefore, the decarboxylase systems could also function in reversible manner wherein the direction of operation depends on the cation gradient and free energy change under the conditions of the physiological steady state [23]. Additionally, regulation of citric acid biosynthesis in bacteria is governed by anabolic and catabolic pathways at the *anaplerotic node* comprising of phosphoenol pyruvate-oxaloacetate-pyruvate [24] which makes any targeted genetic modification unstable. Nature and number of the enzymes involved at the *anaplerotic node* in fluorescent pseudomonads vary in different strains reflecting their catabolic diversity. Thus, it is necessary to understand the effect of alterations carried out at the *anaplerotic node* in different strains for universal application of the system.

Present study demonstrates the selection of suitable NADH insensitive *cs* and citrate transporter genes for citric acid secretion and developing plasmid transformants and genomic integrants containing artificial citrate operon of the selected genes under *lac* promoter in several fluorescent pseudomonads. The effect of plasmid transformants and genome integrants on citric acid secretion and MPS ability demonstrates that genetic modifications can be effective strategy even in strains with variable metabolic traits.

## Materials and Methods

### Bacterial Strains, Plasmids and microbiological methods

The bacterial strains and plasmids used in this work are summarized in Table 1. All *E. coli* strains were grown in Luria-Bertani medium [25] at 37 ^°^C unless otherwise stated. Media were supplemented with ampicillin (100 µg ml^−1^), chloramphenicol (30 µg ml^−1^); kanamycin (50 µg ml^−1^), gentamycin (50 µg ml^−1^) and tetracycline (20 µg ml^−1^) as required. For routine biochemical characterization, *Pseudomonas fluorescens* and its recombinants were grown on M9 minimal medium supplemented with a micronutrient solution and iron with glucose [0.5% (w/v)], as the carbon source as well as TRP buffered medium (with 75 mM Tris HCl, pH 8.0) containing 75 mM glucose. For the minimal media the concentrations of the antibiotics were reduced to one fourth of the concentrations added to the rich media mentioned above. Cultures were incubated at 30°C and shaken on an orbital platform (Orbitek, Scigenics Biotech, Chennai, India) operating at 200 strokes per minute. All strains were stored at −20°C in MicroBank vials and subcultured not more than twice prior to experimentation. Agar (1.5% w/v) was added to solidify the bacteriological media when necessary before sterilization. Aerobic growth was carried out under shaking in Erlenmeyer flasks containing 0.1 volumes of medium. Growth was measured spectrophotometrically at 600 nm.

**Table 1:** Bacterial strains and plasmids used in this study.

Strains/Plasmids	Description	References/Source
***E. coli*** ** Strains**		
*E. coli* DH5α	F-φ80Δ*lacZ*ΔM15Δ(*lacZYA*-*argF*) U169 *recA1endA1 hsdR17* (*rk^−^*, *mk^+^*) *phoAsupE44* λ-*thi-1gyrA96 relA1*, host for general cloning	[25]
*E. coli* DH5α pYCInt	*E. coli* DH5α containing *yc* operon in mini Tn7 delivery plasmid AKN69 under p*lac*	This study
*E. coli* W620	CGSC 4278 *glnV44 gltA6 galK30* LAM-*pyrD36relA1 rpsL129 thi^−^* ^1^, Str^r^	*E. coli* Genetic Stock Center, Yale University, USA.
SM101 AKN68	*E. coli* SM101::λpir helper plasmid pUXBF13 providing the Tn7 transposes proteins Amp^r^	A generous gift of Prof. Soren Molin, Technical University of Denmark, Denmark
JM105 AKN69	*E. coli* JM105 containing miniTn7 (Gm) PA1/04/03-eyfp-a	A generous gift of Prof. Soren Molin, Technical University of Denmark, Denmark
***Pseudomonas*** ** strains**		
*P. fluorescens* PfO-1	Agricultural soil isolate	[48] A generous gift of Dr. Mark Sylvi, Tufts University School of Medicine, Boston, Massachusetts USA
*P. fluorescens* Pf-5	Isolated from plant roots; Biocontrol properties	[49] A generous gift of Dr. L. Thomashow Washington State University, Pullman, Washington USA
*P. fluorescens* CHAO-1	Isolated from Swiss soil; Biocontrol properties.	[50] A generous gift of Dr. L. Thomashow Washington State University, Pullman, Washington USA
*P. fluorescens* ATCC13525	Wild type	[13] MTCC, Chandigarh, India
*P. fluorescens* Fp315	Native isolate from wheat rhizosphere	[13] A generous gift of Dr. B. N. Johri, G. B. Pant University of Agriculture and Technology, Pantnagar, India
*P. fluorescens* P109	Native isolate from wheat rhizosphere	[13] A generous gift of Dr. B. N. Johri, G. B. Pant University of Agriculture and Technology, Pantnagar, India
**Plasmids**		
pGM160	Shuttle vector for *Streptomyces* and *E. coli*, Ap^r^, Gm^r^,	[44]
pUCPM18	Broad host range cloning vector Amp^r^	[45]
pUCPM18Gm	pUCPM18 containing gentamycin resistance gene,	This study
pAB8	pUCPM18 with *npt*II gene, Amp^r^/Km^r^	[12]
pAB7	*gltA* gene of *E. coli* under p*lac* promoter in pAB8, Amp^r^/Km^r^	[12]
pY145F	pAB8 carrying *gltA1* (containing mutation of Y145F in *gltA*) gene under p*lac* promoter, Amp^r^ Km^r^	This study
pR163L	pAB8 carrying *gltA2* (containing mutation of R163L in *gltA*) gene under p*lac* promoter, Amp^r^ Km^r^	This study
pK167A	pAB8 carrying *gltA3* (containing mutation of K167A in *gltA*) gene under p*lac* promoter, Amp^r^ Km^r^	This study
pCitC	pUCPM18Gm carrying *Salmonella typhimurium*sodium citrate transporter (*citC*) gene under p*lac* promoter, Amp^r^ Gm^r^	This study
pCitM	pUCPM18Gm carrying *Bacillus subtilis* Mg^2+^ citrate transporter (*citM*) gene under p*lac* promoter, Amp^r^ Gm^r^	This study
pYC	pUCPM18Gm carrying both *gltA1* and *Salmonella typhimurium citC* genes under p*lac* promoter, Amp^r^ Gm^r^	This study

Amp^r^, Ampicillin resistance; Gm^r^, Gentamycin resistance; Km^r^, Kanamycin resistance; Cm^r^, Chloramphenicol resistance; Str^r^, Streptomycin resistance.

### Molecular Biology techniques

All molecular biology procedures such as plasmid extraction, transformation and *in vitro* manipulations of DNA, PCR amplification were carried out according to standard protocols [25]. *Taq* DNA polymerase and its buffer, dNTPs, PCR primers and other enzymes for DNA *in vitro* manipulations were obtained from Genei Merck Pvt. Ltd., Bangalore, India and Sigma Chemicals Pvt. Ltd., Bangalore, India, respectively, and were used according to manufacturer's instructions.

### Construction of plasmids containing CS and Citrate transporter and artificial citrate operon

The *E. coli* NADH insensitive citrate synthase (CS) encoding gene *glt*A, with three different modifications viz *gltA1*, *gltA2* and *gltA3* were obtained as 1281 bp fragments flanked by *Sac*I and *Bam*HI sites by PCR amplification of plasmid DNA carrying the respective genes using pairs of primers: 5′C***GAGCTC*** *GGGCCC*TTTTTCACGGAGGAAACCACAATG GCT GAT ACA AAA GC 3′ and 5′CG***GGATTC*** *CGGATCCG*TTA ACG CTT GAT ATC GC 3′. Ribosomal binding site corresponding to *Pseudomonas* sp. has been added (underlined) while restriction enzymes *Sac*I and *Kpn*I in forward and *Apa*I and *Bam*HI site in reverse of *cs* primers (bold and italicized) were added. The appropriately digested fragments were then inserted in to *Sac*I/*Bam*HI site within the pAB8 multiple cloning site (MCS). pY145F, pR163L and pK167A plasmids containing the NADH insensitive *gltA* genes *gltA1*, *gltA2* and *gltA3*, respectively, in the correct orientation with respect to the *lac* promoter of pAB8 were selected by restriction enzyme mapping and PCR amplification using gene specific primers.

A 1.3 kb *Hin*dIII fragment of pGM160 plasmid containing gentamycin resistance gene was inserted at *Hin*dIII site of pUCPM18 plasmid to obtain pUCPM18Gm and used as a cloning vector for the citrate transporter genes. A 1281 bp DNA fragment containing *citC* gene was amplified from *S. typhimurium* genomic DNA with primer pair: GG***GGGCCC*** CG***GGATCC***CGCACGGAGGAATCAACTT ATG ACC AAC ATG ACC CAG GCT TC {5′ primer, *Apa*I/*Bam*HI bold italicized, ribosomal binding site (RBS)underlined} and 5′ GG***GGTACC*** CCGC***TCTAGA***GC TTA CAC CAT CAT GCT GAA CAC GAT GC (3′ primer, *Kpn*I/*Xba*I bold italicized). Similarly, a 1341 bp DNA fragment containing *citM* gene was obtained from *B. subtilis* genomic DNA using primer pair: 5′ GG***GGGCCC***CG***GGATCC***CGCACGGAGGAATCAACTT ATG TTA GCA ATC TTA GGC TTT CTC ATG ATG(5′ primer, *Apa*I/*Bam*HI bold italicized, RBS underlined) and 5′ GG***GGTACC*** CCGC***TCTAGA***GC TTA TAC GGA AAT AGA GAT CGC ACC G (3′ primer, *Kpn*I/*Xba*I bold italicized). *Taq* DNA polymerase and its buffer, dNTPs and primers were obtained from Bangalore Genei Pvt. Ltd., India and Sigma Chemicals Pvt. Ltd., Bangalore, India, respectively, and were used according to manufacturer's instructions. The *citC* gene was blunted and inserted into *Eco*RV site of pBluescript KS and further subcloned in *Xba*I site in the MCS of pUCPM18Gm to generate pcitC. The *citM* gene was cloned into pTZ5/7R using InsT/Aclone PCR Product Cloning Kit, MBI Fermentas, (pJE4) and subcloned into *Eco*RI-*Sal*I site in the MCS of pUCPM18Gm to generate pcitM plasmid. The presence of the appropriate plasmid was checked by PCR using gene specific primer and M13 primer and by restriction enzyme mapping. To construct the artificial citrate (*yc*) operon 1281 bp *gltA1* gene was excised from pY145F with *Sac*I-*Bam*HI and ligated to the upstream of *citC* gene in pcitC yielding pYC.

### Functional complementation study of gene constructs in *E. coli*

Functional expression of *gltA* genes was checked by complementation of glutamate auxotrophy of *E. coli* W620 CS mutant grown in minimal medium supplemented with IPTG (0.1 mM) and thiamine (100 mg/ml) with and without glutamate (340 mg/ml). Functionality of citrate transporters was monitored by growth of *E. coli* DH5α transformants in Koser citrate broth supplemented with IPTG (0.1 mM).

### Development of *P. fluorescens* transformants harbouring the recombinant plasmids

*P. fluorescens* PfO-1 was independently transformed with NADH insensitive plasmids by using the NaCl/CaCl_2_ method [26] to obtain the recombinant strains expressing *E. coli* NADH insensitive *cs* gene. pAB7 and pAB8 transformants were used as a control in all experiments. *P. fluorescens* PfO-1 strains co-expressing the citrate transporter and *gltA1* genes (pYF*CitC* or pYF*CitM*) were developed by transforming both the plasmids independently or as an operon in same plasmid to obtain pYC (with CitC transporter). The single and double transformants were selected on *Pseudomonas* agar containing accordingly kanamycin, gentamycin or both. pUCPM18Gm and pAB8 transformants were used as controls for all the experiments.

### Genomic integration and PCR analysis of chromosomal insertion of citrate operon

For genomic integration, the citrate operon along with constitutive *lac* promoter was amplified using constitutive p*lac* forward and *citC* reverse primers. The 2.7 kb fragment containing p*lac*-*gltA1*-*citC* operon was blunted and inserted into the *Sma*I site of integration delivery plasmid AKN69 to generate pYC-Int plasmid. Prior to cloning the chloramphenicol resistance gene of the plasmid was disrupted using *Pvu*II. The clone was confirmed by double digestion with *Eco*RI-*Xba*I restriction site and PCR. The construct along with the helper pUXBF13 plasmid containing the transposase genes were delivered into *P. fluorescens* strains by the electroporation method [27]. Electroporation was carried out using BioRad electroporator (Bio-Rad Laboratories Chennai (India) Pvt. Ltd.) with the following settings: 25 µF, 200 Ω, 2.5 kV (the time constant should be <5 ms). Preliminary confirmation of the genomic integrants were done by monitoring the growth on gentamycin plate, fluorescence and reconfirmed by PCR using the primer pair: 5′ATATCGACCCAAGTACCGCC (Tn7Gm, nt 509 from the start site of *aaC*1 gene) and 5′**CG** ***GGATTC*** *C* ***GGATCC*** *G*TTAACGCTTGATATCGC (3′ primer of *gltA* gene).

### Physiological experiments

Fresh cultures grown with overnight shaking at 30°C in 3 ml Luria–Bertani broth were aseptically harvested, washed thrice with sterile 0.85% (w/v) saline and resuspended in 1 ml of the same under sterile conditions. The resultant bacterial cell suspension was used to inoculate M9 minimal medium supplemented with 100 mM glucose and micronutrient cocktail to give an initial cell density of OD_600_ 0.01–0.03 (∼2.6×106 c.f.u./ml). 75 mM Tris-Cl (pH 8.0) buffered medium with rock phosphate (TRP) was used to represent P deficient medium supplemented with 75 mM glucose. Inoculum for TRP medium was prepared in M9 medium as described earlier [28] and an initial cell density of 1.4×10^8^ cfu/ml was maintained. Batchculture studies were performed by shaking 150 ml conical flasks with 30 ml of the inoculated medium on an Orbitek rotary shaker (Orbitek, Scigenics Biotech, Chennai, India) at 30°C and 200×g.

### Analytical techniques

Change in cell density (OD_600_ nm) using Helios γ spectrophotometer (Thermo Spectronics, Cambridge, UK) was considered as the measure of growth while drop in pH of the media was taken as the measure of acid production. The observations were continued till the medium pH reduced to less than 4 or O.D at 600 nm was greater than 2.1 ml Samples drawn at regular intervals were centrifuged at 9,200×*g* for 1 min at 4°C, filtered through 0.2 µm nylon membrane (MDI Advanced Microdevices, India) and used to measure residual glucose enzymatically (using the GOD-POD kit, Enzopak, Reckon Diagnostics Pvt. Ltd, India) and organic acid analysis using HPLC (Varian Microsorb RP-18 column) operated at room temperature using mobile phase of 0.01 M Na_2_HPO_4_ +5% acetonitrile at a flow rate of 0.2 ml min^−1^, column effluents were monitored using a UV detector at 210 nm. The physiological parameters like growth rate, specific glucose depletion rate and biomass yield were calculated as described earlier [28]. Organic acid yields were expressed as grams of organic acid formed per gram of glucose depleted per gram of dry cell weight. Graph Pad Prism (version 3.0) for Windows, Graph Pad Software, San Diego, CA, USA (http://www.graphpad.com) and Microsoft Excel (Microsoft, 2003 Microsoft Excel computer software, Richmond, WA) were used for statistical analysis of the parameters. Each parameter has been represented as mean ± SD or mean ± SEM as specified in the legends to figures and tables. One-way ANOVA and Bonferroni's multiple comparison tests were performed for all treatments using Graph Pad prism software.

### Preparation of cells/cell free extracts and enzyme assays

Citrate Synthase (CS, 4.1.3.7), Isocitrate dehydrogenase (ICDH, 1.1.1.42), glucose-6-phosphate dehydrogenase (G-6-PDH, EC 1.1.1.49) were assayed using both mid log and late log-stationary phase cultures whereas Isocitrate lyase (ICL, 4.1.3.1), glucose dehydrogenase (GDH) and pyruvate carboxylase (PYC) were assayed in late log to stationary phase. Preparation of cell free extracts and enzyme activities for G-6-PDH, PYC, ICDH and ICL assays as well as whole cell suspension for GDH assay were carried out as described earlier [12], [28]. All enzymes were assayed at room temperature (30±2°C) against appropriate controls without the substrate and enzyme source in the reaction mixture. Total protein concentration of the crude extract and whole cell suspensions was measured by a modified Lowry method [29] using bovine serum albumin as standard, along with appropriate corrections to rule out the interference of Tris [30]. One unit of specific enzyme activity was defined as the amount of protein required to convert 1 nmole of substrate per minute.

### Mineral phosphate solubilization (MPS) ability

The dicalcium phosphate solubilizing ability of wild type and *gltA* transformants of fluorescent pseudomonads were tested on Pikovskaya's agar [31]. Three microliters of the bacterial inoculums prepared as described above, was aseptically spotted on the agar plates and was allowed to dry completely followed by incubation at 30°C. Preparation of bacterial inoculum for phosphate-solubilization studies was similar to that for the abovementioned physiological experiments. Rock phosphate solubilizing ability was monitored using 75 mM Tris buffered (TRP) minimal medium with 75 mM glucose. P-solubilization on Pikovskaya's agar was determined by monitoring the phosphate solubilization index (PSI) whereas drop in the medium pH from 8.0 to <5 on TRP broth indicated by formation of a red zone on buffered Senegal rock phosphate. Methyl Red was used in agar plates to observe rock phosphate solubilization. The P liberated from rock phosphate in the TRP medium was measured by the Ames method [32].

## Results

### Overexpression of citrate synthase, transporter and artificial citrate operon in *P. fluorescens* PfO-1

**Figure 1:**
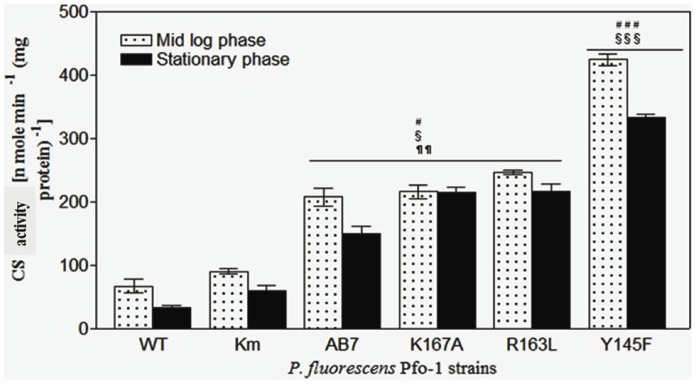
Citrate synthase activity of *P. fluorescens* PfO-1 transformants. The activity was estimated with transformants containing pAB8 (vector control, denoted as Km), pAB7 (denoted as AB7), pK167A, pR163L and pY145F along with the untransformed native strain (WT). Cultures were grown on M9 minimal medium with 100 mM glucose to mid log or stationary phase. Activity is represented in the units of nmole/min/mg total protein. The values are depicted as Mean ± S.E.M of 4 independent observations. # Comparison of parameters with WT, § with respect to vector control AB8, ¶ comparison of parameter between Y145F and AB7/R163L/K167A. ###, §§§, ¶¶¶: P<0.001; ##, §§, ¶¶: P<0.01; #, §, ¶: P<0.05.

**Figure 2:**
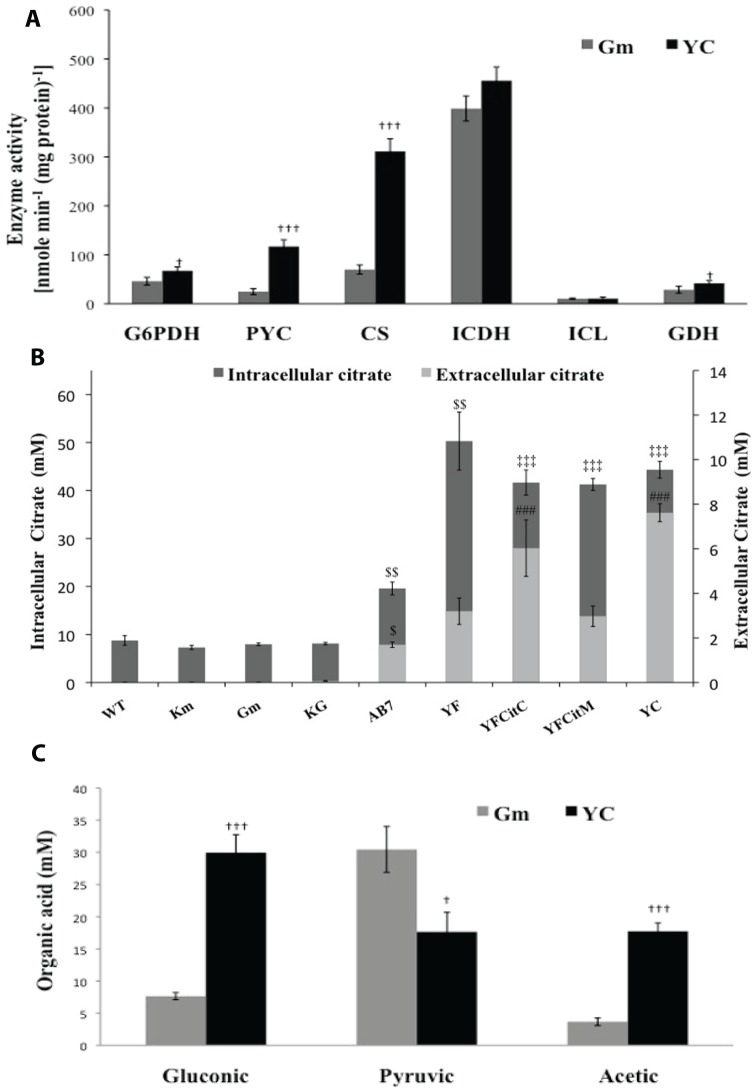
Organic acid profile and enzyme activities of *P. fluorescens* PfO-1 transformants. Activities of key enzymes (A) using stationary phase cultures grown on M9 medium with 100 mM glucose were represented in the units of nmoles/min/mg total protein. Intracellular and extracellular citric acid levels (B) are represented in grey bars and black bars respectively. Gluconic-pyruvic-acetic acid levels (C) and citric acid levels were estimated from stationary phase cultures grown on same media. Results are expressed as Mean ±S.E.M of 4 independent observations. WT is native untransformed strain, Gm indicates vector control containing pUCPM18Gm, Km indicates vector control containing pAB8 and YC indicates tansformant with artificial citrate operon pYC; KG indicates vector control with both the pUCPM18 and pAB8 plasmids cotransfromed; YF indicates transformant with pY145F; YFCitC and YFCitM indicate cotransformants carrying plasmid pY145F along with pCitC and pCitM, respectively. * comparison of parameters with wild type control; $ comparison of parameters with vector control pAB8, # comparison between parameters of AB7 and YF.***,$ $ $,###: P<0.001; **,$ $,##: P<0.01; *,$,#: P<0.05.

Three gene variants of the *gltA* gene, encoding NADH insensitive CS were cloned independently in broad host range vector pUCPM18Gm under *lac* promoter and the functionality was confirmed by complementation of glutamate auxotrophy in *E. coli* W620 (**Figure S1**). Amongst three *cs* mutants, *P. fluorescens* PfO-1 (pY145F) showed highest CS activity of 424.6±16.1 U and 333.4±8.5 U in the mid log and stationary phase, respectively, on M9 minimal medium which is about 4.7 and 5.6 fold higher than that the control strain *Pf* (pAB8) (**Figure 1**). Hence, for further constructs of artificial citrate operon this mutant gene was used. The transformant accumulated 50 mM citrate intracellularly and secreted 3.2 mM (**Figure 2B**). However, the specific growth rate, specific total glucose utilization rate and total amount of glucose utilized after 30 h remained unaffected. The amount of glucose consumed intracellularly was reduced by 1.49 fold in *Pf* (pY145F) as compared to *Pf* (pAB8). The increase in CS activity in *Pf*(pY145F) strain improved the biomass yield by 3.2, 2.38 and 2 fold compared to WT, *Pf* (pAB8) and *Pf* (pAB7), respectively (**Table 2**).

**Table 2:** Physiological variables and metabolic data for *P. fluorescens* PfO-1 transformants grown on M9 minimal medium with 100 mM glucose.

Bacterial strains			Growth parameters		
	Specific growth rate µ (h^−1^)†	Total glucose utilized(mM) ‡	Glucose consumed (mM) ‡	Biomass yield (Ydcw/Glc† (g g^−1^)	Specific glucose utilization rate QGlc† [g (g dcw)^−1^ h^−1^]
WT	0.42±0.03	50.45±7.08	45.94±6.62	0.12±0.02	4.5±0.55
Pf(pAB8)	0.59±0.04	69.88±9.4	62.22±8.5	0.16±0.03	7.2±1.3
Pf (pUCPM18Gm)	0.52±0.01	54.23±4.76	46.54±4.49	0.12±0.03	6.6±0.85
Pf (pAB7)	0.65±0.04	65.86±4.86	48.6±3.57	0.19±0.04	6.35±1.56
Pf (pY145F)	0.61±0.06	71.46±6.7	41.71±5.75^$^	0.38±0.01^$^	6.8±1.03
Pf (pYC)	0.53±0.02	67.47±6.97	37.52±7.59^†^	0.31±0.053^†^	7.01±0.45

The results are expressed as the mean±SEM of readings from four independent observations. †Determined from mid-exponential phase of each experiment. ‡Determined at the time of pH drop (30 h). $, † P<0.001, as compared to respective controls.

*E. coli* DH5α transformants of *citC* and *citM* transporter cloned in *Pseudomonas* stable vector pUCPM18Gm could grow on Koser citrate broth medium containing citrate as a sole carbon source upon induction with 0.1 mM IPTG, demonstrating the functionality of the respective protein (data not shown). pYC plasmid was constructed by incorporating the *cs*Y145F and *citC* genes both under the *lac* promoter in pUCPM18Gm plasmid as an artificial citrate operon. After confirming the functionality in *E. coli cs* mutant by growth on citrate (**Figure S2**), the plasmid was incorporated in *P. fluorescens* PfO-1. Extracellular citric acid levels in *Pf*(pYC) increased by 2.3 and 84.6 folds as compared to *Pf* (pY145F) and *Pf*(pAB8) strains, respectively (**Figure 2B**). Corresponding extracellular citrate yield increased by 2.07 fold and 76 fold compared to respective controls. Thus the efficient expression of the genes encoding citrate producing enzyme under the *lac* promoter provided *P. fluorescens* with the ability to secrete citric acid. Stationary phase culture supernatents of *Pf* (pYC) showed 3.91 and 4.81 fold elevated gluconic and acetic acid levels, respectively, with concomitant 1.73 fold decreased pyruvic acid level as compared to *Pf*(pGm) (**Figure 2C**).

In *Pf* (pYC), the periplasmic GDH activity increased by 1.46 fold as compared to the *Pf* (pGm) in late log to stationary phase of growth. Similarly, a significant increase in G6PDH, PYC and CS activities by 1.46, 4.74 and 4.5 fold, respectively, were observed as compared to the controls. However, ICL and ICDH activities in the stationary phase cultures remained unaltered (**Figure 2A**). Significant pH drop was found in case of *Pf* (pYC) compared to wild type and vector control strain. Specific growth rate, specific total glucose utilization rate after 30 h remained unaffected. Total amount of glucose utilized increased by 1.44 fold and glucose consumption is reduced by 1.27 fold in *Pf* (pYC) as compared to *Pf* (pGm). *Pf* (pYC) strain had improved the biomass yield by 2.58 fold compared to *Pf* (pGm) (**Table 2**).

### Growth and biochemical characterization of diverse *P. fluorescens* genomic integrants containing artificial citrate operon

**Figure 3:**
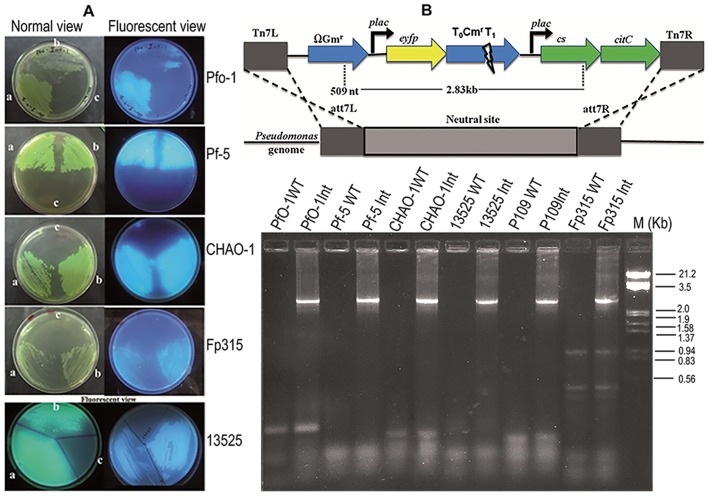
Construction of *P. fluorescens* genomic integrants carrying artificial citrate operon. Natural fluorescence and antibiotic resistance of genetically modified *P. fluorescens* strains (A); *P. fluorescens* pYC plasmid transformants (a), *P. fluorescens* genomic integrants (b) and *P. fluorescens* wild type (c) grown on *Pseudomonas* agar plates containing ampicillin and gentamycin. Schematic representation of the genomic region of *Pseudomonas* genome showing the integrated mini transposon and gel picture showing confirmation of integrants by PCR amplification of genomic DNA isolated from *P. fluorescens* wild type and genomic integrants using Tn7-Gm (510 nt from the start site of Gm^r^ gene) and *cs* reverse primer (PCR product∼2800 bp) (B).

Artificial citrate operon was integrated into six *P. fluorescens* strains using Mini-Tn7 transposon system. The integrants were selected by fluorescence and gentamycin resistance (**Figure 3A**). Presence of *yc* operon in the genome was confirmed by PCR amplification of 2.8 kb using specific primers (**Figure 3B**).

The growth characteristics of *P. fluorescens yc* operon genomic integrants were determined upon growth under buffered-RP (TRP, 75 mM Tris-Cl pH 8.0) broth minimal medium containing 75 mM glucose. All genomic integrants had growth equivalent to wild type and plasmid transformant. On the other hand, *P. fluorescens* Fp315 genomic integrants showed an enhanced growth rate by 1.6 fold as compared to the plasmid transformants while growth rate of *P. fluorescens* Pf5 plasmid transformants was drastically reduced (1.7 fold) as compared to the wild type culture and genomic integrants (**Table S1**). Genomic integrants of *P. fluorescens* PfO-1 and Fp315 strains acidified the medium pH below 5 within 48 h while other strains required 72–96 h.

*P. fluorescens* CHAO1genomic integrants showed an increase in total amount of glucose utilized by 1.36 fold and 1.25 fold as compared to the wild type and plasmid transformant, respectively, whereas the *P. fluorescens* P109 genomic integrant showed a 1.3 fold decrease in the total glucose utilization.Total glucose utilization remains unaltered in *P. fluorescens*PfO-1, Pf5 and ATCC 13525 integrants. The amount of glucose consumed decreased by 1.57 fold and 1.3 fold as compared to wild type and *Pf*(pYC) in *P. fluorescens*PfO-1 genomic integrant, respectively. Similar decrease was observed in *P. fluorescens* Pf5, ATCC13525 and Fp315 strains by 2.5, 1.26 and 2.26, respectively, as compared to wild type strain. On the other hand, an increased glucose consumption by 1.26 fold and 3 fold were observed in ATCC13525 and P109 genomic integrant, respectively, as compared to wild type strain. Glucose consumption remain unaltered in *P. fluorescens* CHAO1 genomic integrant. The biomass yield and specific glucose utilization rate were remained unaltered in *P. fluorescens* CHAO1 and ATCC13525 genomic integrants. Biomass yield was increased by 3.5, 2.2, 2.1 and 1.7 fold in *P. fluorescens* PfO-1, Pf5, P109 and Fp315, respectively, as compared to wild type control. *P. fluorescens* Fp315 genomic integrant showed a decrease in specific glucose utilization rate by 1.44 fold whereas 1.96 fold increase in specific glucose utilization rate were monitored in Pf5 as compared to the wild type control. All these variations in results are shown in **Table S1**.

**Figure 4:**
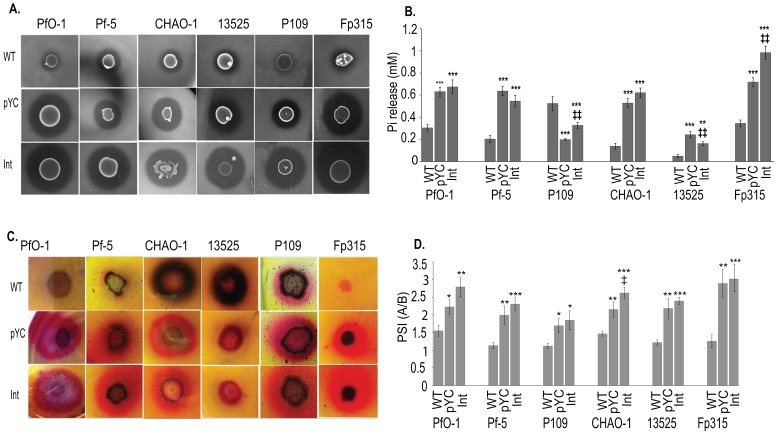
Effect of *P. fluorescens yc* operon genomic integrants on citric acid accumulation, citric and gluconic acid secretion. Intracellular citric acid levels (A), extracellular citric (B) and gluconic (C) acid levels (mM) estimated from the stationary phase cultures of wild type (WT), plasmid transformants containing *yc* operon (pYC), *yc* operon genomic integrants (Int) of different *P. fluorescens* strain PfO-1, Pf5, CHAO1, ATCC13525 (13525), P109 and Fp315 grown in TRP minimal medium for 96–120 h. Results are expressed as mean ± S.E.M of 4–8 independent observations, † comparison of parameters with WT; ‡ comparison of parameters between plasmid transformants (pYC) and genomic integrants (Int). †††, ‡‡‡: P<0.001; ††,‡‡: P<0.01; †,‡: P<0.05.

On TRP mediumcontaining 75 mM glucose, *P. fluorescens* CHAO1, ATCC13525 and Fp315 plasmid transformants and integrants, intracellular citrate level was increased upto 4.6 fold as compared to the control strain (**Figure 4A**). However, the intracellular citrate levels were found to be similar in both genomic integrant and plasmid bearing strains. The CS activity correlated with the intracellular citrate levels. *P. fluorescens* Pf5(Int) and P109(Int) showed a similar increase in intracellular citrate level by 3.5 and 3.27 fold, respectively, as compared to the control. Exception of this effect was PfO-1(Int), in which the intracellular citrate level is 1.3 fold less as compared to PfO-1(pYC). This data when compared with the respective plasmid bearing strain showed a significant reduction in citrate level by 1.27 and 1.5 fold, respectively. An enhancement of extracellular citrate levels was achieved in both plasmid bearing and genomic integrants of *P. fluorescens* strains when compared to their respective control *Pf* (pGm) strain (**Figure 4B**). All the strains harboring citrate operon showed enhanced gluconic acid secretion up to 2 fold as compared to the vector control except *Pf*(P109) where decreased secretion was monitored (**Figure 4C**). Genomic integration of citrate operon improved the gluconic secretion in *Pf* (CHAO1) and *Pf* (Fp315) as compared to their respective plasmid transformants.

The effect of genomic integration of p*lac yc* operon on the key enzymes involved in periplasmic direct oxidation, intracellular phosphorylative pathway and anaplerotic reactions were monitored in *P. fluorescens* (**Table 3**). GDH activity increased upto 2.7 fold in PfO-1(Int), Pf5(Int), CHAO1(Int) and Fp315(Int) as compared to the respective vector control strain whereas significicant decrease in GDH activity was observed in ATCC13525(Int) (1.4 fold) and P109(Int) (2.76 fold). Improvement of GDH activity monitored in PfO-1(Int), Pf5(Int), CHAO1(Int) as compared to their respective plasmid control hence correlating with gluconic acid secretion. Reduction in G6PDH activity upto 50%was observed in both PfO-1 (Int) and PfO-1(pYC) as compared to control with exceptions in CHAO1 strain which showed a significant enhancement of G6PDH activity by 3 fold as compared to the vector control. The percentage change in G6PDH activity was correlated with change in GDH activity. Thus, the citrate operon genomic integrants showed the diversion of glucose flux more towards the direct oxidation pathway by increase in GDH activity with concommitant decrease in G6PDH activity as well as glucose consumption. However strain wise variation was also monitored.

**Table 3:** Activities of key glucose catabolic enzymes in *P. fluorescens* strains wild type (WT), plasmid transformants harbouring *yc* operon, Int (genomic integrants of *yc* operon) grown in TRP broth (pH 8.0) minimal media.

P. fluorescens strains	Enzyme Activity^a^					
	CS	G6PDH	ICDH	PYC	ICL	GDH
PfO-1WT	66.87±5.8	234.42±4.42	233.56±12.4	7.48±0.72	3.11±0.44	34.02±7.36
PfO-1 Pyc	177.1±18.3^†††^	118.22±5.08^†††^	204.99±7.84	38.26±2.93^†††^	1.35±0.22	54.41±5.43
PfO-1Int	212.9±16^†††^	114.23±7.35^†††^	224.16±9.38	37.13±3.78^†††^	5.28±0.93	76.30±8.55^†††,‡^
Pf5-1WT	37.2±3.66	223.88±20.4	140.66±11.75	16.72±2.78	1.5±0.17	54.65±7.76
Pf5-1 pYC	171.99±7.12^†††^	19.18±3.53^†††^	239.96±25.75^††,‡^	12.01±2.55	1.27±0.16	91.33±4.4^††^
Pf5-1Int	175.15±8.34^†††^	25.77±5.16^†††^	162.56±28.3	11.74±1.57	4.01±0.56^†††,‡‡‡^	103.20±9.09^††,‡‡‡^
CHAO-1WT	38.1±3.98	41.56±4.17	261.36±13.07	10.13±2.42	2.36±0.16	27.10±4.89
CHAO-1 pYC	103.2±8.53^†††^	24.67±4.85^††^	278.25±11.5	47.20±14^††^	2.45±0.22	23.54±3.68
CHAO-1Int	158.5±16.1^†††,‡‡^	123.59±6.65^†††,‡‡‡^	261.98±25.45	32.53±5.18	1.76±0.33	74.07±10.65^†††,‡‡‡^
13525 WT	38.5±2.55	175.26±9.28	220.03±15.72	4.90±0.66	1.41±0.24	61.06±6.07
13525pYC	234.3±10.86^†††^	90.58±7.82^†††^	233.43±17.55	90.58±3.42^††^	1.18±0.21	66.13±9.26
13525Int	237.9±7.98^†††^	31.80±5^†††,‡‡‡^	230.06±11.99	31.80±2.94†	1.58±0.32	43.53±6.61^‡^
P109 WT	75.9±9	158.56±32.97	156.86±23.89	158.56±4.76	1.38±0.24	87.08±11.98
P109pYC	165.9±4.95^†††^	117.96±15.36	180.46±13.53	117.96±2.59	2.08±0.34	26.24±8.26^†††^
P109 Int	141.9±4.68^†††,‡^	126.02±9.42	191.83±12.87	126.02±5	1.73±0.26	37.51±5.6^†††^
Fp315 WT	63.8±7.26	42.22±7.95	256.8±12.89	42.22±2.41	2.37±0.35	35.57±6.6
Fp315pYC	167.5±5.2^†††^	23.18±4.92†	263.92±15.98	23.18±5.14	2.07±0.18	73.84±9.09^†††^
Fp315 Int	217.4±8.08^†††^	27.37±4.69	245±22.63	27.37±4.4	2.77±0.49	79.16±8.39^†††^

a Activities are expressed as nmoles/min/mg total protein from mid-log to stationary phase cultures and are given as mean±SEM of readings from four independent observations.

† Comparison of parameters with WT, ‡ comparison of parameters between pYC and Int. †††, ‡‡‡: P<0.001; ††, ‡‡: P<0.01; †, ‡: P<0.05.

All genomic integrants showed no alteration in ICDH and ICL activities with an exception of Pf5 (pYC) and Pf5 (Int) which showed an increase in ICDH activity by 1.7 fold and ICL activity by 2.7 fold, respectively, as compared to wild type strain. Interestingly, majority of genomic integrants of *yc* operon showed CS activity at par with the plasmid transformants. The CS activity is increased by 3.17 and 2.64 fold in *P. fluorescens*PfO-1*yc* operon genomic integrant and plasmid bearing strains, respectively. Similarly, the increase was 4.73 and 4.6 fold in Pf5, 4.1 and 2.7 fold in CHAO1, 6 fold each in ATCC13525, 1.9 and 2.2 fold in P109 and 3.4 and 2.65 fold in Fp315 as compared to the respective wild type controls. Remarkably, the CHAO1 genomic integrant showed 1.5 fold higher CS activity than that of CHAO1 (pYC) strain. PYC activity remain unaltered in *P. fluorescens*Pf5, CHAO1, P109 and Fp315 genomic integrants whereas an increase by 5 fold and 2.8 fold in *P. fluorescens* PfO-1(Int) and ATCC13525(Int), respectively, were observed compared to control (**Table 3**).

### MPS ability of *P. fluorescens yc* transformants and genomic integrants

**Figure 5:**
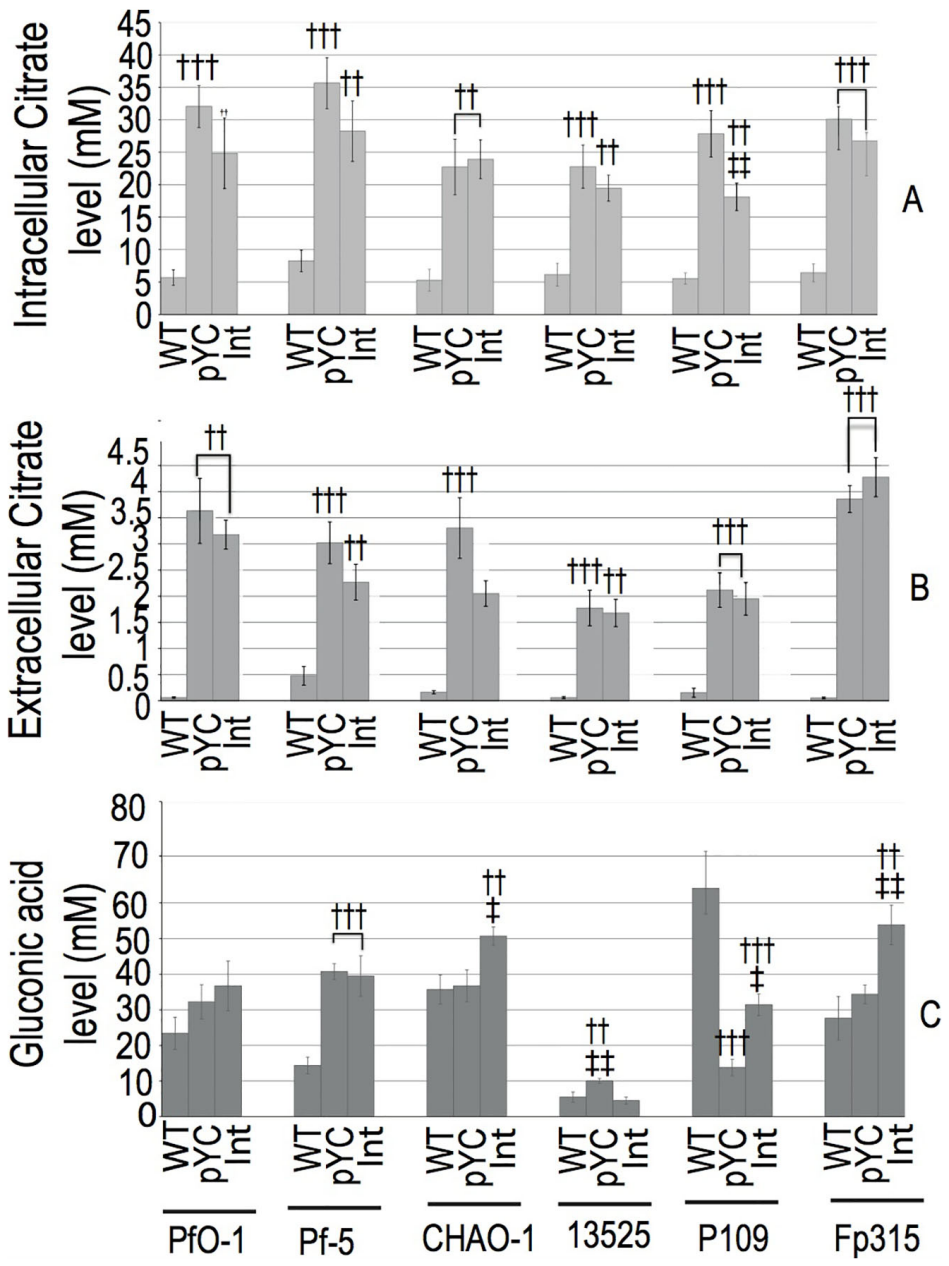
Phosphate solubilisation activities of *P. fluorescens yc* operon genomic integrants and plasmid transformants. Zone of clearance and PSI (Phosphate solubilisation index) (A and B) in Pikovskaya's agar medium during 96–120 h of growth. Zone of colouration (C) and Pi release (D) of transgenic *P. fluorescens* strains in TRP broth and TRP agar medium during 96–120 h of growth. WT: wild type strain; pYC: *P. fluorescens* with pYC plasmid; Int: *P. fluorescens yc* operon genomic integrant. Results are expressed as Mean ±S.E.M of 4 independent observations. * Comparison of parameters with wild type control; ‡ comparison between parameters of plasmid transformants (pYC) and genomic integrants (int).***, ‡‡‡: P<0.001; **,‡‡: P<0.01; *,‡: P<0.05.

All *yc* bearing strains, monitored on both Pikovskaya's agar and TRP agar media, showed significant improvement on solubilization index as compared to their respective wild type control. Genomic integrants *P. fluorescens* PfO-1 and Fp315 *yc* operon showed maximum dicalcium phosphate solubization ability (**Figure 5A and 5B**). Solubilization index of both the strains varied in the order of WT<pGm <pYF <pYC <Int*yc*.

On TRP medium, all cultures showed an improvement in the zone of coloration as compared to the wild type (**Figure 5C**). Moreover, the genomic integrants of PfO-1, Pf5 and Fp315 showed faster rock phosphate solubilization as evident from the appearance of zone of coloration within 48–72 h of growth as compared to 96–120 h of plasmid transformants and wild type (Data not shown). Except Fp315 wild type, none of the wild type cultures could show zone of coloration on TRP medium containing 75 mM Tris HCl (pH 8.2) and 75 mM glucose. All *P. fluorescens yc* operon plasmid transformants and genomic integrants showed significant enhancement of Pi release as compared to the wild type strains with an exception of P109 wherein compared to the wild type no improvement of Pi release was observed. *P. fluorescens* Fp315 genomic integrant showed a maximum Pi release of 983.4±58.78 µM which was even 1.37 fold higher as compared to plasmid transformants. In contrast, ATCC13525 genomic integrant showed a decrease in Pi level by 1.5 fold as compared to the plasmid transformants (**Figure 5D**). The Pi release among the *P fluorescens* genomic integrants was in the increasing order of ATCC13525<P109 <Pf5 <CHAO1 <PfO-1<Fp315.

## Discussion

In present study, metabolic engineering was employed to enhance citrate productionin *Pseudomonas fluorescens* to increase the mineral phosphate solubilization efficiency. In an earlier investigation, *P. fluorescens* ATCC13525 overexpressing *E. coli* wild type *cs* gene (allosterically inhibited by NADH) reported to increase CS activity by 2 fold with accumulation of 18.3 mM intracellular citrate [12]. Since native CS enzyme is subject to allosteric inhibition by NADH, it was hypothesized that over-expression of NADH insensitive variants of the CS would lead to further improvement of the citrate production in the genetically modified strains. Three site-directed allosteric *cs* mutants were reported with decreased affinity of NADH binding in the order R163L*<<*K167A <<Y145F with a maximum Ki value of 790±210 µM for Y145F as against wild type CS (2.8±0.4 µM) [14]. In the present study, overexpression of these NADH insensitive *gltA* mutants in *P. fluorescens*PfO-1 and demonstrated increased CS activity in the order of pAB8<<pAB7≤R163L≤K167A <Y145F (**Figure 1**) and citrate accumulation in the order of pAB8<<pAB7<R163L<K167A <Y145F (**Figure 2B**) suggesting that enhanced citric acid biosynthesis in *P. fluorescens* PfO-1 is directly correlated with loss of NADH inhibition of CS enzyme. Earlier studies of overexpression of phosphoenolpyruvate carboxylase (*ppc*) and *gltA* genes alone and together showed that CS regulates citrate biosynthesis [13]. In addition, present study demonstrates that NADH mediated inhibition of CS activity regulates the flux of TCA cycle in *P. fluorescens*. Remarkably, the higher citrate accumulation in *Pf* (pY145F) did not hinder growth. Our study is supported by the fact that the mechanisms for pH sensing and cytoplasmic pH homeostasis enable most bacteria to tolerate or grow at pH values that are outside the cytoplasmic pH range they must maintain for growth [33].

Although extracellular citrate levels were enhanced in *Pf* (pY145F) as compared to *Pf* (pAB7), the amount secreted corresponds to only 6% of the intracellular level (**Figure 2B**), suggesting limitation of the bacterial ability to secrete the acid into the extracellular medium. *P. fluorescens* possesses H^+^-dependent citrate transporter [34], [35], which normally supports their growth on citrate as a sole carbon source. Our results suggest that this transporter seems to be inefficient in the efflux of citrate as also observed in our previous studies with the wild type citrate synthase gene [12]. The active pH homeostasis favors this in mesophilic bacteria [36]. The active transport system for citrate excretion appears to be the main rate-determining factor in citrate overproduction by yeasts [37]. Present study demonstrates that *S. typhimurium* CitC transporter is able to enhance the citrate excretion and is more efficient than *B. subtilis* CitM transporter suggesting that Na^+^ ion gradient is much more favorable than Mg^+2^ ions for citrate efflux in fluorescent pseudomonads.

Plasmid dependent metabolic load reported to cause alterations in the central carbon metabolism in *P. fluorescens* 13525 and lose in MPS ability in *Enterobacter asburiae* PSI3 [38], [39]. Our study has also included genome integration instead of plasmid mediated expression to study the effect of the additional copy of *cs* and citrate transporter gene on metabolism avoiding the plasmid load effect. Genomic integration of citrate operon in six different *P. fluorescens* strains showed enhanced CS activity and citric acid secretion. This effect turned out to be similar or even better for genomic integrants compared to mid log to stationary phase cultures from plasmid transformants containing the same operon. This result suggests that the metabolic load of maintenance of the plasmid counteracts the beneficial effect of increased copy number. Overexpression of polyphosphate kinase (*ppk*) gene using low copy mini F plasmid was reported to cause a 20-fold increase in polyphosphate (poly P) content in the early stage of growth whereas the enhancement was 80% in the stationary phase [40]. On the other hand, profile using multicopy plasmid depicted a different behavior wherein PolyP increased dramatically within the first 3 h, but the polyP levels at 24 h were low and similar to that of low-copy plasmid.

The influence of metabolic diversity of catabolically versatile fluorescent pseudomonads overexpressing citrate operon was reflected in the qualitative and quantitative changes in their metabolism. Glucose catabolism in fluorescent pseudomonads consists of direct oxidative and phosphorylative pathways that determine the glucose utilization whereas TCA cycle flux is controlled at the anaplerotic node towards anabolic and oxidative phosphorylation pathways depending on the availability of electron acceptors. Variations in different strains arise from the nature and number of enzymes participating in these pathways especially at the *anaplerotic* node [24]. In our study, all genomic integrants showed similar effects but differed in a few parameters. Increased gluconic acid levels with concomitant reduction in pyruvic acid levels and increased PYC activity indicate probable diversion of pyruvate flux towards increased OAA biosynthesis in response to increased CS activity. Even in *A. niger* the enhancement of anaplerotic reactions replenishing TCA cycle intermediates predisposes the cells to form high amounts of citric acid [41]. Enhancement of biosynthetic reactions due to shortage of TCA cycle intermediates was also observed in citric acid accumulating *E. coli* K and B strains in the form of increased glyoxylate pathway [42], [43]. However in our study, similar increase in flux through glyoxylate shunt was not apparent as evident from very low and unaltered ICL activity (**Table 3**). Low ICL activity was consistent with earlier reports in *P. fluorescens* ATCC13525 and *P. indigofera* in which ICL contributed negligibly to glucose metabolism [12], [44]. Enhanced CS activity also increased the periplasmic glucose oxidation, which is reflected, by increase in GDH activity and gluconic acid production.

Overexpression of citrate operon enhanced direct oxidative pathway, phosphorylative pathway but decreased TCA cycle flux and glyoxylate pathway in all fluorescent pseudomonads. Enhanced citric acid accumulation had no effect on anabolic process as evident from similar generation time of the bacteria (**Table 2**). No change in the generation time of the transformants also suggests that the increased ATP generation via direct oxidative pathway appear to be coupled with decrease in ATP generation mediated by TCA flux.

Incorporation of citrate operon enhanced secretion of total citric and gluconic acids in all fluorescent pseudomonads that was correlated with enhanced MPS ability in both Pikovskaya's and TRP medium. Similar artificial citrate operon also enhanced P solubilization efficacy of *Herbaspirillum seropedicae* Z67 [45] and in *Enterobacter hormaechei* DHRSS [46]. The amount of citric acid secreted was similar in *P. fluorescens* PfO1 and *E hormaechei* DHRSS, but significantly lower in *H. seropedicae* Z67. However, genetically modified *H. seropedicae* Z67 enhanced rice plant growth. It will be interesting to know the effectiveness of *Pseudomonas* genomic integrants in supplementing P to plants. Recently, *P. fluorescens* 13525 containing an artificial oxalate operon was demonstrated to secrete oxalate and demonstrated good MPS ability in TRP and alfisols along with enhancement of mung bean plants grown in alfisols [47]. Thus, metabolic engineering approaches could be useful in developing effective PSMs.

## Supporting Information

Figure S1 **Complementation of** ***E. coli*** **W620 mutant phenotype by wild type and NADH insensitive** ***cs*** **plasmids.** WT *E. coli* W620 represents *E. coli* W620 deletion mutant of *cs* gene pR163L, pK167A and pY145F represent plasmids containing NADH insensitive *cs* genes. All plasmid bearing strains were induced with 0.1 mM IPTG. Growth was monitored on M9 minimal medium with 0.2% glucose. +/− at the top of each image indicates presence and absence of respective supplements in the media.
(TIF)

Figure S2 **Growth of** ***E. coli*** **DH5α on Koser's citrate broth.** *E. coli* DH5α containing pGm and pYC plasmids grown on media containing citrate as sole carbon source. All plasmid bearing strains are supplemented with 100 mg/ml thiamine, antibiotic 1/4th of the recommended dose and without supplementation of glutamate. +/− at the top of each image indicates presence and absence of respective supplements in the media.
(TIF)

Table S1 **Physiological variables from** ***P. fluorescens*** **transformant and integrant strains grown on75 mM glucose in TRP minimal medium.** Growth rate (µ), Biomass yield (BMY) and specific glucose utilization rate (Q Glc) were estimated from mid log phase cultures and total glucose depleted (TGD) and glucose consumption (GC) was determined at the time of pH drop (96 h). The values are depicted as Mean ± S.E.M of 4 (N = 4) independent observations. †Comparison of parameters with vector control Gm, ‡comparison of parameters between pYC plasmid transformants and genomic integrants of fluorescent pseudomonads. †††, ‡‡‡: P<0.001; ††, ‡‡: P<0.01, †, ‡: P<0.05.
(DOCX)
