# Supplementary figures and images for: Artificial Citrate Operon Confers Mineral Phosphate Solubilization Ability to Diverse Fluorescent Pseudomonads
Source: PLoS One. 2014 Sep 26;9(9):e107554. doi: 10.1371/journal.pone.0107554 (PMC4178029; doi:10.1371/journal.pone.0107554)

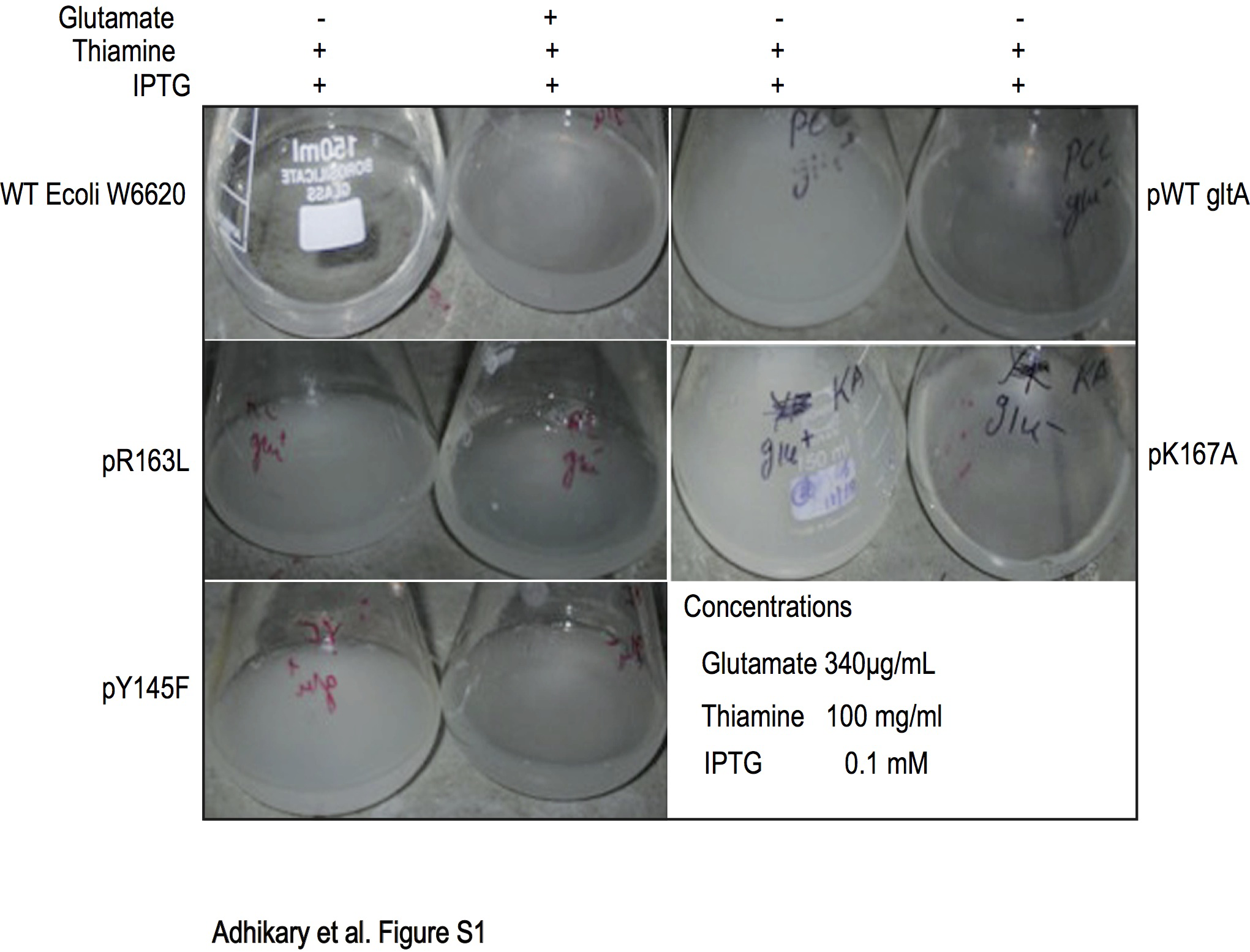

Supplement: Figure S1 — Complementation of E. coli W620 mutant phenotype by wild type and NADH insensitive cs plasmids. WT E. coli W620 represents E. coli W620 deletion mutant of cs gene pR163L, pK167A and pY145F represent plasmids containing NADH insensitive cs genes. All plasmid bearing strains were induced with 0.1 mM IPTG. Growth was monitored on M9 minimal medium with 0.2% glucose. +/− at the top of each image indicates presence and absence of respective supplements in the media. (TIF) [file pone.0107554.s001.tif]

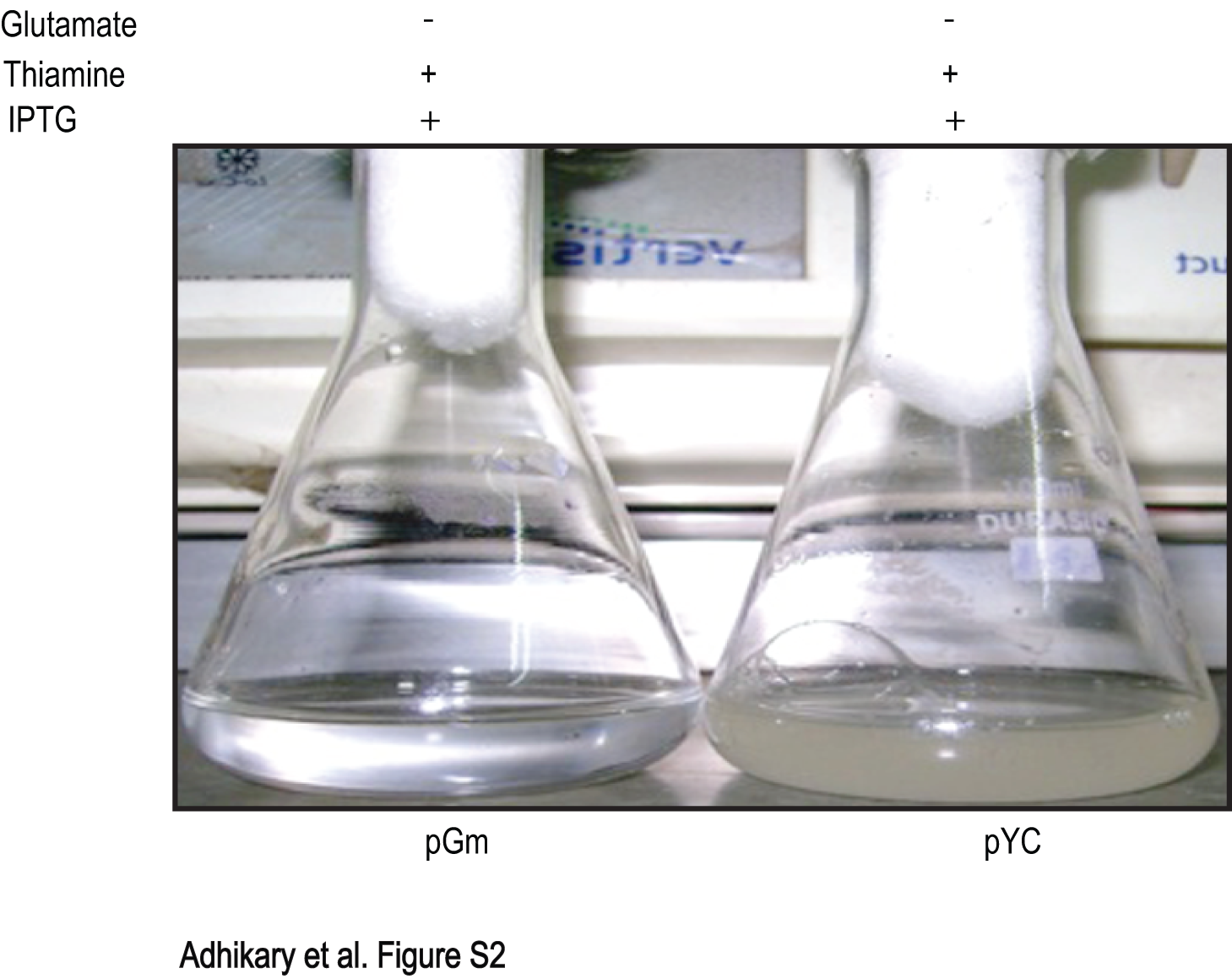

Supplement: Figure S2 — Growth of E. coli DH5α on Koser's citrate broth. E. coli DH5α containing pGm and pYC plasmids grown on media containing citrate as sole carbon source. All plasmid bearing strains are supplemented with 100 mg/ml thiamine, antibiotic 1/4th of the recommended dose and without supplementation of glutamate. +/− at the top of each image indicates presence and absence of respective supplements in the media. (TIF) [file pone.0107554.s002.tif]
