# Supplementary material for: Artificial Citrate Operon Confers Mineral Phosphate Solubilization Ability to Diverse Fluorescent Pseudomonads
Source: PLoS One. 2014 Sep 26;9(9):e107554. doi: 10.1371/journal.pone.0107554 (PMC4178029; doi:10.1371/journal.pone.0107554)
Supplement: Table S1 — Physiological variables from P. fluorescens transformant and integrant strains grown on75 mM glucose in TRP minimal medium. Growth rate (µ), Biomass yield (BMY) and specific glucose utilization rate (Q Glc) were estimated from mid log phase cultures and total glucose depleted (TGD) and glucose consumption (GC) was determined at the time of pH drop (96 h). The values are depicted as Mean ± S.E.M of 4 (N = 4) independent observations. †Comparison of parameters with vector control Gm, ‡comparison of parameters between pYC plasmid transformants and genomic integrants of fluorescent pseudomonads. †††, ‡‡‡: P<0.001; ††, ‡‡: P<0.01, †, ‡: P<0.05. (DOCX) [file pone.0107554.s003.docx]

| **Parameter** | **PfO-1** | | | **Pf-5** | | | **CHAO-1** | | | **ATCC13525** | | | **P109** | | | **Fp315** | | |
| --- | --- | --- | --- | --- | --- | --- | --- | --- | --- | --- | --- | --- | --- | --- | --- | --- | --- | --- |
|  | **WT** | **YC** | **Int** | **WT** | **YC** | **Int** | **WT** | **YC** | **Int** | **WT** | **YC** | **Int** | **WT** | **YC** | **Int** | **WT** | **YC** | **Int** |
| **μ (h^-1^)** | **0.24±0.035** | **0.29±0.05** | **0.31±0.02** | **0.27±0.04** | **0.16±0.02^†^** | **0.22±0.04** | **0.28±0.03** | **0.23±0.02** | **0.3±0.03** | **0.21±0.05** | **0.19±0.03** | **0.17±0.02** | **0.26±0.04** | **0.21±0.02** | **0.22±0.02** | **0.34±0.04** | **0.3±0.03** | **0.47±0.05^‡^** |
| **TGD (mM)** | **54.68±6.59** | **58.89±3.3** | **56.06±6.68** | **70.9±3.42** | **67.15±4.3** | **61.6±5.05** | **47.2±3.65** | **51.06±3.79** | **64.0±5.55^‡^** | **49.3±4.23** | **44.2±4.34** | **39.13±5.2** | **70.2±4.4** | **45.75±3.78^†††^** | **52.56±4.03^††^** | **62.14±4.35** | **50.66±3.76^†^** | **70.27±3.7^‡‡^** |
| **GC (mM)** | **30.6±2.91** | **25.16±2.35** | **19.2±2.21 ^‡‡^** | **55.8±4.2** | **26.32±3.6^†††^** | **22.38±3.16^†††^** | **11.5±2.17** | **14.52±2.53** | **13.5±3.05** | **43.9±3.9** | **34.7±3.27^†^** | **34.33±2.89^†^** | **6.68±1.45** | **32.21±3.61^†††^** | **20.21±3.31^††,‡‡^** | **34.01±3.87** | **15.16±3.13^††^** | **17.83±3.3** |
| **BMY (g/g)** | **0.075±0.006** | **0.17±0.025 ^††^** | **0.26±0.016 ^†††, ‡‡^** | **0.06±0.01** | **0.1±0.02** | **0.13±0.02^†^** | **0.16±0.01** | **0.19±0.01** | **0.21±0.02^†^** | **0.07±0.02** | **0.11±0.01** | **0.079±0.01** | **0.11±0.01** | **0.19±0.02^†††^** | **0.23±0.005^†††^** | **0.21±0.01** | **0.31±0.01^†††^** | **0.36±0.01^†††, ‡‡^** |
| **QGlc (g/g/h)** | **1.79±0.12** | **2.51±0.41** | **1.98±0.45** | **4.11±0.23** | **7.23±0.37^†††^** | **8.08±0.27^†††^** | **3.19±0.23** | **4.52±0.39^†^** | **3.88±0.49** | **2.67±0.37** | **1.23±0.21^††^** | **1.83±0.24^†^** | **1.25±0.06** | **.75±0.26^†††^** | **1.62±0.23^‡‡^** | **3.17±0.35** | **6.53±0.43^†††^** | **4.58±0.34^††, ‡‡^** |

**Table S1**: **Physiological variables from *P. fluorescens*transformant and integrant strains grown on75 mM glucose in TRP minimal medium**.

Growth rate (µ), Biomass yield (BMY) and specific glucose utilization rate (Q Glc) were estimated from mid log phase cultures and total glucose depleted (TGD) and glucose consumption (GC) was determined at the time of pH drop (96 h).

The values are depicted as Mean ± S.E.M of 4 (N=4) independent observations**.** †Comparison of parameters with vector control Gm, ‡comparison of parameters between pYC plasmid transformants and genomic integrants of

fluorescent pseudomonads. †††, ‡‡‡: P< 0.001; ††, ‡‡: P< 0.01, †, ‡: P< 0.05
